# Parental work absenteeism is associated with increased symptom complaints and school absence in adolescent children

**DOI:** 10.1186/s12889-017-4368-7

**Published:** 2017-05-12

**Authors:** Mari Hysing, Keith J. Petrie, Tormod Bøe, Børge Sivertsen

**Affiliations:** 1 The Regional Centre for Child and Youth Mental Health and Child Welfare, Uni Research Health, Postboks 7810, 5020 Bergen, Norway; 2 Department of Psychological Medicine, University of Auckland, Auckland, 1142 New Zealand; 3 Department of Health Promotion, Norwegian Institute of Public Health, Bergen, Norway; 4 Department of Research and Innovation, Helse Fonna HF, Haugesund, Norway

**Keywords:** School attendance, Symptom complaints, Adolescence, Parental work absence

## Abstract

**Background:**

Previous studies have proposed that having parents out of work may influence adolescent illness behaviour and school attendance. However, prior research investigating this question has been limited by retrospective reporting and case control studies. In a large epidemiological study we investigated whether parental work absence was associated with symptom complaints and increased school absenteeism in adolescents.

**Methods:**

We analysed data from a large epidemiological study of 10,243 Norwegian adolescents aged 16–19. Participants completed survey at school, which included demographic data, parental work absence and current health complaints. An official registry provided school attendance data.

**Results:**

Parental work absence was significantly related to the number of adolescent symptom complaints as well as school absenteeism. Having a father out of work was associated with an increased likelihood of being in the highest quartile of symptom reporting by an odds-ratio of 2.2 and mother by 1.6 (compared to the lowest quartile). Similarly, parental work absenteeism was associated with an increased likelihood of being in the highest quartile for school absence by an odds-ratio of 1.9 for a father being out of work and 1.5 for a mother out of work. We found that the number of adolescent symptom complaints mediated the relationship between parental work absenteeism and school absenteeism.

**Conclusion:**

We found that parental work absence was significantly associated with the number of adolescent symptom complaints and school absenteeism. The results suggest that parents may play a critical modelling role in the intergenerational transmission of illness and disability behaviour.

## Background

A number of studies have speculated that parental sickness and work absence (here defined as unemployment, sick leave or permanent work disability) may play a key role in influencing both adolescent symptom reporting and school absence. Several studies in the chronic pain literature have noted a relationship between the existence of parental pain models at home and the reporting of both pain symptoms in young people and the disruption to their normal daily activities due to pain [1–3]. Having a parent with a pain condition seems to put children at risk of increased illness behaviour and somatic complaints [4].

A similar pattern has been noted by clinicians and researchers working in the area of children’s gastrointestinal complaints and unexplained pain. Here researchers have also noted a link between parental pain and symptom complaints and functional gastrointestinal symptoms or pain in their children [5, 6]. In an illustrative case control study, Campo and colleagues [7] compared mothers of 8–15 year children and adolescents presenting with unexplained abdominal pain to a control group of mothers presenting for routine care. The researchers found that compared to control mothers, mothers of children and adolescents with unexplained abdominal pain were significantly more likely to have a history of irritable bowel syndrome, migraine and somatoform disorders, as well as have been more frequent users of ambulatory health care services.

To date there has been little work looking at the association between the impact of parental illness on children and, specifically, the role of parental work absence on symptom complaints and school absenteeism [8]. Interpretation of the results of previous studies in the area has been weakened by the use of retrospective reporting. In these studies participants were asked about their current symptoms and asked to remember whether parents had pain conditions when they were growing up (e.g. (3)). Other case control studies have drawn their sample from patients already in clinical care. Clearly, more data is needed from large population studies where data about adolescent symptoms and school absence can be collected concurrently with parental work absence. In the current study we drew on a large general population survey of adolescents collected in Western Norway to investigate whether parental work absence was associated with a greater level of symptom complaints in adolescence. A second aim was to explore if parental work absence was associated with adolescent school absence and if this was mediated by adolescent symptom complaints.

## Methods

### Procedure and participants

In this population-based study from 2012, we used data from the youth@hordaland-survey of adolescents in the county of Hordaland in Western Norway. The general aim of the youth@hordaland-survey was to assess mental health, lifestyle, school performance and the use of health-service in adolescents. In collaboration with the Hordaland County, all adolescents born between 1993 and 1995 and all students attending secondary education were invited to participate. The adolescents received information about the study via their official school e-mail, and one school class (about 45 min during regular school hours) was allocated for them to complete the Internet based questionnaire. A teacher was present to organize the data collection and to ensure confidentiality. Those not attending school received the survey by mail posted to their home address. Survey staff were available by phone for both the adolescents and school personnel to answer queries related to the research. The adolescents’ parents were informed about the study, while the adolescents themselves consented to participating in the study, as Norwegian regulations state that individuals aged 16 years and older are required to provide their own consent. The study was approved by the Regional Committee for Medical and Health Research Ethics in Western Norway. A total of 19,439 adolescents were invited to participate in the survey, of which 10,254 agreed, yielding a participation rate of 53%.

### Measures

#### Sociodemographical characteristics

Gender and date of birth were identified through the personal identity number in the Norwegian National Population Register.

#### Parental work absenteeism

Parental work absence was defined by an open-ended question where the adolescents described their parents work status for mothers and fathers separately. This was coded according to ISCO-08 classification [9]. Being in work was defined as parents with a work ISCO code or parents reported to be students (mothers, 8558). The out-of-work category consisted of parents that were not in work (including parents on sick leave, disability pensions, as well as unemployed parents and homemakers (mothers, *n* = 594). Parents that were dead (mothers, *n* = 33; fathers, *n* = 55) or retired (mothers, *n* = 4; fathers, *n* = 64) were coded as missing.

#### Adolescent symptom complaints

Symptom complaints were measured using 5 items, four from the HBSC-symptoms checklist [10]. Participants reported the frequency of headache, abdominal pain, back pain, dizziness and pain in neck/shoulders, experienced during the last 6 months on a five-point scale ranging from “more or less every day” to “seldom or never”. The 5 items were summed to a total score (range 0–20), and then divided in 4 categories by quartiles. In addition, a count variable was created summing the number of *different* symptoms the adolescents reported experiencing “weekly” or more often (range 0–5).

#### Adolescent school absence

Non-attendance at school over the past semester (6 months) was assessed using official register-based data provided by Hordaland County Council. For the purpose of the present study, we used both the continuous variable (mean of days and hours), as well as categorical variables using the quartiles as cut-offs based on the continuous variable.

### Statistics

IBM SPSS Statistics 22 for Mac (SPSS Inc., Chicago, Ill) was used for all regression analyses. Multinomial logistic regression analyses were conducted to examine the predictive effect of the parental work absenteeism (independent variables) on 1) increased symptom complaints and 2) school absence in the adolescent children (dependent variables). Multinomial logistic regression analyses were also used to examine the association between number of weekly symptoms (independent variable) and level of school absence (dependent variable).

In order to assess whether adolescent symptom complaints mediated the association between parental work affiliation and school absence, indirect effects analyses were conducted within a structural equation modelling framework using version 0.5–16 of the Lavaan package [11] in R for Mac version 3.1.1 [12]. Maximum likelihood estimation with bootstrapped (*k* = 1000) standard errors and confidence intervals was used according to recent recommendations [13]. Incomplete responses were handled using pairwise deletion, ensuring high data retention in the analyses (92.6% in analyses with mothers and 88.4% in analyses with fathers).

## Results

### Parental work absenteeism and symptom complaints

Parental work absenteeism was significantly associated with increased symptom complaints in adolescent children. As detailed in Table 2, having a father that did not work was associated with an increased odds-ratio of 2.2 for reporting a symptom level in the 4th quartile (compared to the 1st quartile). A similar, but somewhat weaker effect was found for mothers not working (OR = 1.6). Parental work absenteeism was also associated with an increased odds of reporting less severe symptoms levels, with ORs of reporting symptom levels in in the 3rd quartile of 1.4 for both mother and father not working (see Table 1 for details).

**Table 1:** Parental work absenteeism associated with increased symptom complaints in adolescent children

		Symptom complaints (quartiles)			
		3rd quartile		4th quartile	
	% (n)	OR	95% CI	OR	95% CI
Parent not working					
Father not working	3.7% (320)	1.43	1.00–2.04	2.24	1.1–3.11
Mother not working	7.1% (647)	1.44	1.14–1.81	1.63	1.30–2.05

Reference: lowest (1st) quartile

### Parental work absenteeism and school absence

Parental work absenteeism was also significantly associated with increased school absence in adolescent children (Table 2). Having a father that did not work was associated with an increased odds-ratio of 1.9 for having a school absence in the 4th quartile (7 days or more of school non-attendance). A similar, but somewhat weaker effect was found for mothers not working (OR = 1.5). The associations were present for both *days* and *hours* of school absence (see Table 2 for details).

**Table 2:** Parental work absenteeism associated with school absence in adolescent children

	Days of school absence			
	3rd quartile (4–6 days)		4th quartile (7 ≥ days)	
	OR	95% CI	OR	95% CI
Parent not working				
Father not working	1.01	0.70–1.46	1.89	1.40–2.57
Mother not working	1.06	0.83–1.35	1.49	1.20–1.85
	Hours of school absence			
	3rd quartile (4–9 h)		4th quartile (10 ≥ hours)	
	OR	95% CI	OR	95% CI
Parent not working				
Father not working	1.68	1.18–2.39	1.99	1.41–2.82
Mother not working	1.31	1.01–1.68	1.76	1.38–2.24

Reference: lowest (1st) quartile

### Adolescent symptom complaints and school absence

**Fig. 1:**
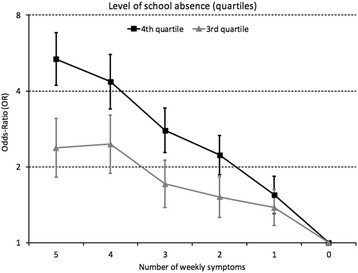
Weekly symptom complaints associated with school absence (3rd and 4th quartiles compared to 1st quartile) among adolescents in the youth@hordaland-study. *Markers* represent odds-ratios (OR) and *error bars* represent 95% confidence intervals (Y-axis has a logarithmic scale)

**Fig. 2:**
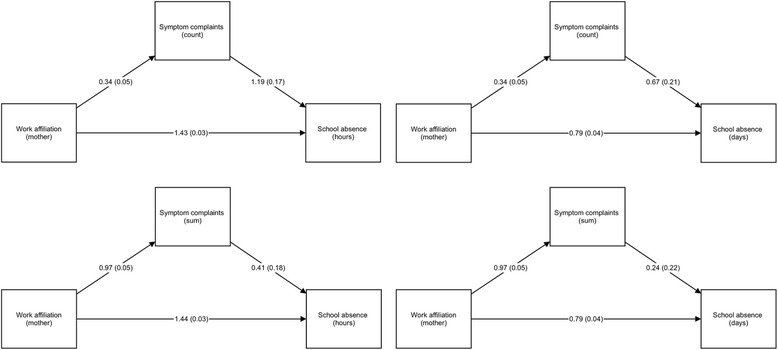
Path models illustrating symptom complaints (as sum/count) acting as a mediator of the association between maternal work affiliation and school absence (in hours/days). Unstandardized coefficients and standardized coefficients (in parenthesis) shown

The number of weekly symptom complaints was associated with increased odds of school absence in a dose-response manner (Fig. 2). For example, compared to reporting no symptoms, having 5 different weekly symptoms increased the odds of substantial school absence (4th quartile) by 5.4, whereas reporting only 1 symptom was associated with an OR of 1.5. A similar trend was found for absence in the 3rd quartile (see Fig. 1 for details).

### Symptom complaints as mediators of the association between parental work absenteeism and school absence

**Fig. 3:**
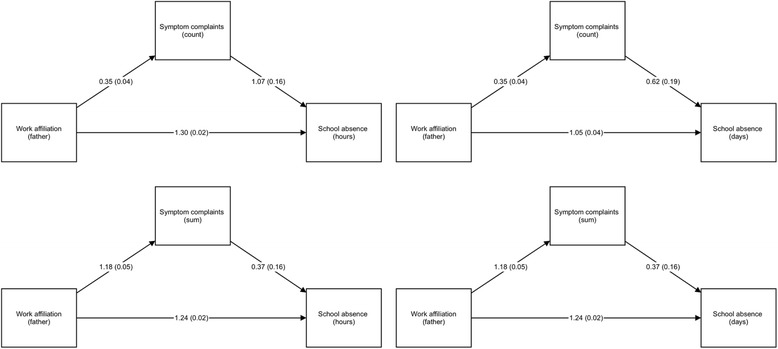
Path models illustrating symptom complaints (as sum/count) acting as a mediator of the association between paternal work affiliation and school absence (in hours/days). Unstandardized coefficients and standardized coefficients (in parenthesis) shown

The relationship between parental work affiliation and hourly absence from school was mediated by symptom complaints, as depicted in Figs. 2 and 3. In regression models, parental work absenteeism was significantly associated with increases in school absence and with increased symptom complaints, and symptom complaints were associated with school absence. Symptom complaints mediated 17–26% of the total effects on school absence, and the models explained 2.5–5.3% of the variance in school absence, see details in Table 3.

**Table 3:** Tests of the indirect effects of symptom complaints (as count and sum) on the relations between parental work affiliation and school absence (in days and hours)

Parent work affiliation	Measure of symptom complaints	Measure of School absence	Estimate and 95% CI for total effects *b* (upper, lower)	Estimate and 95% CI for indirect effects *b* (upper, lower)	Percent of total effect mediated %	Explained variance of school absence *R* ^*2*^
Mother	Count	Hours	1.836^***^ (0.807–2.944)	0.405^***^ (0.241–0.600)	22.25	0.031
Mother	Count	Days	1.020^***^ (0.527–1.566)	0.227^***^ (0.133–0.331)	22.06	0.046
Mother	Sum	Hours	1.836^***^ (0.835–2.857)	0.400^***^ (0.238–0.583)	21.79	0.033
Mother	Sum	Days	1.020^***^ (0.524–1.563)	0.234^***^ (0.137–0.343)	22.94	0.053
Father	Count	Hours	1.669^**^ (0.619–2.962)	0.370^***^ (0.164–0.596)	22.17	0.025
Father	Count	Days	1.266^***^ (0.572–1.967)	0.214^***^ (0.098–0.340)	16.90	0.040
Father	Sum	Hours	1.669^**^ (0.460–2.816)	0.431^***^ (0.235–0.673)	25.82	0.026
Father	Sum	Days	1.266^***^ (0.579–1.966)	0.264^***^ (0.134–0.401)	20.85	0.046

Bootstrapped (*k* = 1000) confidence intervals shown

^**^ *p* ≤ .01, ^***^ *p* ≤ .001

## Discussion

The findings in the present study from a general population study of Norwegian adolescents show that parental work absence was significantly associated with both the number of adolescent symptom complaints and school absence. While co-occurring adolescent symptom complaints accounted for some, but not all of this association, a mediation analysis confirmed both a direct and significant indirect effects via adolescent symptom complaints on their school absence.

The results suggest a strong interrelation between parents and adolescent absence behavior, as well the importance of health complaints in understanding this association. The results confirmed our hypothesis of parental work absence being associated with both symptom reports and school absence in the adolescents. The association between parental and adolescents school absence in relation to health complaint is in line with previous reports from clinical studies concerning the association between symptom complaints and responses to health complaints among parents and children [1, 2]. A previous register-based study also found an increased risk of award of disability pension among young adults among parents who are outside the workforce [14]. The association between parental work affiliation, and adolescent health complaints and school absence may thus be one pathway that can account for the intergenerational transmission of disability pension.

From a theoretical perspective, social learning theory may be a useful conceptual framework to understand these associations. Social learning theory posits that learning occurs through observation and vicarious reinforcement of others [15]. In this case, health complaints and school absence in the adolescents may be perceived as a possible response when observing parents with similar attributes and behaviors [16]. As such, it is likely that having a parental role model that is not working may be an important contributing factor for the reported symptom complaint and school absence behavior. From this perspective, absence from work life and school may also be viewed as illness behavior or a coping mechanism in response to health complaints that is learned by adolescents. The intergenerational transmission of illness behavior that is described in the clinical literature may give us a framework to understand these results [5]. The present study is an epidemiological study that is restricted from describing the internal familial processes that contribute to health complaints and school absence in families with parents out of the work force. Other potential pathways may also be at work. Adolescent stress or mental health problems may also be related to both the health complaints and school absenteeism, and may thus constitute alternative contributing factors [17].

Some researchers have hypothesized that parental influence on adolescents may be less in the late adolescents years, when peers influence exerts more influence [18]. Others have advocated a family perspective and-intergenerational perspective to understand the association between health and educational attainment [19], as well as the intergenerational transmission of both health and work affiliation. The associations between parental work absenteeism and both symptom report and school absence in the present study lends support from the family perspective and suggest the continuing strong influence of parents in this age group.

Somatic complaints are frequent during adolescence and associated with a high rate of school absence. This is in line with the functional impairment of somatic complaint in adults, known to be one of the most frequent causes of works absenteeism [20]. We know that health complaints increase during adolescence and become more settled in adulthood [21]. Thus the functional impairment in relation to somatic adolescent complaints may also be a risk factor maintaining a stable future work affiliation and the adoption of future disability payments.

### Strengths and limitations

A major strength of the study is the use of data from administrative registries on school absence and thus reducing the risk of the bias inherent in a simple informant. There are limitations that should be noted. First, parental absence was based on adolescent report and is thus not confirmed by registries, and we do not have information on the parents’ health status. Second, response rate could affect generalizability. Based on previous research from the former waves of the Bergen Child Study, non-participants have been shown to have more psychological problems than participants [22], and it is therefore possible that the level of symptom complaints may be underestimated in the current study. The mean GPA for the participants were not significantly different from the national mean GPA [22]. Thirdly, the cross-sectional nature of the study does not allow for causal inferences, and future studies should investigate if the associations between parental work affiliations and co-occurring school absence will be related to future work affiliation for the adolescents entering adult life, and if the symptom complaints may also explain these associations.

Finally, due to restrictions in statistical power we could not analyze girls and boys separately, and future studies should examine if there at gender specific patterns of association, as has been suggested in studies of early disability when the parent of ones one gender was disabled. [14] Finally, the explained variance of the model is relatively small, and indicates that this is just one of many factors that are related to school absence. Other factors known to be associated with school absence such as for instance sleep problems [23] and alcohol and drug use [24] may also be of importance and were not addressed in the current study.

## Conclusions

In the present study we found parental work absence was significantly associated with the number of adolescent symptom complaints and school absenteeism. The results suggest that parental work models may play a critical role in the intergenerational transmission of illness and disability behaviour. One of the implications of strengthening the understanding of parental influences on both health and school functioning, is the inclusion of a family perspective when health and school personnel meet adolescencents with health complaints and school absence behavior. While the older adolescence is a transitional period with higher degree of independence, the parents still exerts important influence and should be included in interventions.

## Abbreviations

BCS: Bergen Child Study
OR: Odds ratio
